# Matrix metalloproteinase-3 inhibitor retards treadmill running-induced cartilage degradation in rats

**DOI:** 10.1186/ar3521

**Published:** 2011-11-24

**Authors:** Guo-Xin Ni, Li-Qiong Zhan, Mei-Qin Gao, Lei Lei, Yue-Zhu Zhou, Yan-Xia Pan

**Affiliations:** 1 Department of Orthopeadics and Traumatology, Nanfang Hospital, Southern Medical University, Guangzhou Road 1838, 510515, Guangzhou, China; 2 Department of Rehabilitation Medicine, Fujian Medical University, Fuzhou, Fujian, China; 3 Institute of Cancer, Fujian Medical University, Fuzhou, Fujian, China

**Keywords:** running exercise, MMP-3, cartilage, osteoarthritis, inhibitor

## Abstract

**Introduction:**

The effect of intra-articular injection of matrix metalloproteinase (MMP)-3 inhibitor was investigated in a rat model to understand the role of MMP-3 in cartilage degradation induced by excessive loading from running.

**Methods:**

A total of 24 male Wistar rats were randomly assigned into groups of sedentary control (SED), high-intensity running (HIR), HIR + low dosage of MMP-3 Inhibitor I (HIRI1), and HIR + high dosage of MMP-3 Inhibitor I (HIRI2). Rats in the HIR, HIRI1 and HIRI2 groups were intensively trained for six weeks on the treadmill. Those in HIRI1 and HIRI2 groups were provided bilateral intra-articular injections of 80 μL of 0.2 mM and 2 mM MMP-3 Inhibitor I in knee joints once a week, respectively. Blood samples were collected to measure serum MMP-3 level using ELISA. Femoral condyles were collected to observe cartilage characteristics by histochemistry, and MMP-3 as well as collagen II was measured by immunohistochemistry. In addition, cartilage samples were obtained to assess MMP-3 mRNA expression by RT-PCR.

**Results:**

Histological examination showed osteoarthritic changes in rats after six weeks of high intensity running. In comparison to the SED group, significant decreases in glycosaminoglycans (GAG) and collagen content were found in the HIR group, which corresponded to significant increase in serum MMP-3 level, cartilage MMP-3 activity and gene expression. However, such a degradative process was considerably retarded by intra-articular injection of MMP-3 inhibitor at higher dosage. Statistical differences were found between the HIR and HIRI2 groups with regard to GAG and collagen II content, serum MMP-3 level, cartilage MMP-3 activity and gene expression.

**Conclusions:**

High-intensity running for six weeks may lead to cartilage degradation in a rat model. It was shown that the chrondroprotective effect was offered by the use of intra-articular injection of MMP-3 inhibitor. MMP-3 acts as the key mediator of this catabolic change under such mechanical condition. The results also showed that MMP-3 selective inhibitor may be an effective option for retarding such osteoarthritic changes.

## Introduction

While the homeostasis of joints was stabilized in a physiological range of mechanical loading, non-physiological mechanical loading and both overloading and reduced loading, may have deleterious effects, particularly on their cartilaginous components [1-3]. Running is one of the most common weight-bearing activities, and moderate running exercise was found to protect against cartilage degradation in hamsters that could spontaneously develop osteoarthritis (OA) [4]. Nevertheless, excessive running loading was correlated with deleterious effects on cartilage [5-8], hence, an excessive running-induced animal model can serve as a reliable OA model [8].

Excessive mechanical stress can directly damage the cartilage extracellular matrix (ECM) and shift the balance in chondrocytes to favor catabolic activity over anabolism [9]. Catabolism of the cartilage ECM was defined by the occurrence of degradation of both collagen fibrils and proteoglycans [10]. This involves a variety of degradative enzymes, notably matrix metalloproteinases (MMPs), whose basic role is to cleave and initiate the degradation of cartilage components [11]. The MMP family mainly consists of the collagenases (MMPs 1, 8, and 13), which degrade collagen; the gelatinases (MMPs 2 and 9), which target denatured collagen; and the stromelysins (MMPs 3, 7, 10, and 11), which degrade several ECM proteins and are involved in proenzyme posttranslational activation [12].

*In vitro *and *in vivo *studies indicated that a variety of MMPs may be responsive to diverse loading parameters, and may promote degradation of the cartilage collagens and proteoglycan under non-physiological loading conditions [2,7,13-16]. Among the MMPs family, *MMP-3 *appears to be one of the few genes that is up-regulated during the early stage of degeneration [17]. In addition, *MMP-3*-knockout mice showed a 67% reduction in cartilage damage occurring through spontaneous OA [18]. Furthermore, in a rat model, elevated *MMP-3 *activity was coincident with osteoarthritic changes in the knee which were induced by intensive running [7]. Although it is believed that *MMP-3 *may be a key mediator in pathological cartilage matrix degradation, it remains unclear as to its roles in the pathology of cartilage degradation, particularly in which was induced by excessive running loading. All in all, in this current study, a MMP-3 selective inhibitor was administrated to observe whether it offered a protective effect on cartilage degradation induced by excessive running loading and, subsequent to a further understanding of the roles, whether MMP-3 could have a part in the degradation of cartilage ECM under such mechanical conditions.

## Materials and methods

### Experimental animals and study protocol

A total of 24 male Wistar rats (12 to 13 weeks old, weighing 200 to 250 g) were randomly and evenly assigned to one of four groups as follows: 1) sedentary control (SED), 2) high intensity running (HIR), 3) high intensity running + low dosage of MMP-3 Inhibitor I (HIRI1), and 4) high intensity running + high dosage of MMP-3 Inhibitor I (HIRI2). Rats were housed in cages under controlled light/dark (12/12 h) and temperature (22 ± 1°C) conditions, and were provided with food and water *ad libitum*. They were adapted to laboratory conditions for one week before experiments began. The experiment was approved by the animal ethics committee of the institute.

Rats in the HIR, HIRI1 and HIRI2 groups were first accustomed to exercise for one week, by running on a treadmill at the speed of 10 m/minutes for 30 minutes/day. In the subsequent six weeks, they were regularly trained for 1 h/day at the frequency of five days/week, while the speed and inclination of the treadmill were adjusted to 25 m/minute and 12°, which were used to elicit high intensity (85% VO_2_max values) for Wistar rats [19]. Those rats in the HIRI1 and HIRI2 groups were provided intra-articular injections of 80 μL of 0.2 mM and 2 mM MMP-3 Inhibitor I (Chemical formula C_27_H_46_N_10_O_9_S, EMD Biosciences, Inc. San Diego, CA, USA) in knee joints bilaterally once a week, respectively.

### Serum *MMP-3 *concentration

After six weeks, the blood of all rats was collected prior to sacrifice and stored at -80°C until assayed. Serum levels of MMP-3 were measured using commercial enzyme-linked immunosorbent assay kits (ELISA, R & D Systems Inc., Minneapolis, MN, USA) according to the manufacturer 's protocol.

### Tissue preparation

For histological morphology and immunohistochemistry examinations, femoral condyles on the right sides of rats in each group were dissected and fixed in 4% buffered formaldehyde pH 7.4 for 24 hours. Decalcification was completed in 10% EDTA solution, and then the samples were embedded in paraffin wax. Thereafter, they were cut into 5-mm sagittal sections in the medial region.

For the real-time PCR analysis, articular cartilage samples from femoral condyles on the left sides were obtained with a scalpel or rongeur, and flash-frozen in liquid nitrogen at -80°C.

### Histomorphological evaluation

The samples were stained with Safranin-O and histomorphologically evaluated with Mankin grading system, which was previously applied to many experimental OA models [4-8], and proven to be sensitive to early OA changes induced by treadmill exercise [8]. The histological evaluation system for OA was classified into four categories: Mankin score of 0, no OA; Mankin scores of 1 to 5, mild OA; scores of 6 to 10, moderate OA, and scores 11 to 14, severe OA. All sections were graded by two independent observers that were kept unaware of the groups.

Digital densitometry was used for the evaluation of the glycosaminoglycans (GAG) content. For each section, six different areas were digitally captured with a color video camera attached to a light microscope. Illumination intensity and image magnification were kept constant for all images captured. The information was assessed with computer image analysis software (Nikon H600L Microscope and image analysis system, Tokyo, Japan). The values of optical density in six areas were averaged to be GAG content in each section.

### Immunohistochemistry for MMP-3 and collagen type II

In addition to histomorphological evaluation, immunohistological analysis for MMP-3 and collagen type II was performed in all sections. After deparaffinization and rehydration of the tissue sections, MMP-3 and collagen type II were respectively immunostained with the two-step immunohistochemistry method instructed by the manufacturer (Zhongshan Goldenbridge Biotechnology Co., Ltd, Beijing, China).

The sections were incubated with rabbit polyclonal antibody against rat MMP-3 (1:50 dilution, Santa Cruz Biotechnology, Santa Cruz, CA, USA) for 3.5 h at 29°C. The slides were washed in PBS three times, and followed by a 20-minute incubation at 37°C with goat anti-rabbit anti-MMP-3 immunoglobulin G (IgG) (Santa Cruz Biotechnology) and visualized with DAB chromagen. The slides were stained for 40 s, and then we counterstained the nucleus with hematoxylin for 6 s. Negative control sections were prepared with the same protocol above, but primary antibody was replaced by PBS. Immunostaining for MMP-3 in the joint cartilage was evaluated by calculating the ratio of the number of MMP-3 immunoreactive chondrocytes to the total number of chondrocytes within each section.

The procedure of staining collagen type II was similar to the protocol mentioned above with the following changes: monoclonal mouse antirat collagen type II antibody (1:200 dilution, Fisher Scientific, Chicago, IL, USA) was applied as the primary antibody, and anti-mouse IgG/HRP (Fisher Scientific) as the secondary antibody. The collagen type II content was evaluated based on optical density measured using image analysis software (Nikon H600L Microscope and image analysis system).

### Real-time PCR analysis

The samples were frozen in liquid nitrogen, and then were broken into pieces with a masher. The fragments were then mixed and placed in a vessel containing 1 ml Trizol, then centrifuged at 12,000 rpm for 15 minutes at 4°C. Afterwards, 0.2 mL chloroform was added prior to mixing, the supernatant was removed after centrifugation at 12,000 rpm for 15 minutes at 4°C. Then 500 mL isopropanol was added and the samples were once again centrifuged at 12,000 rpm for 15 minutes at 4°C. The supernatant was discarded; 75% ethanol and 500 mL DEPC-treated H_2_O were added. The samples were centrifuged at 7,000 rpm for five minutes at 4°C, the supernatant was discarded, and the pellet was air dried. Subsequently, 30 mL of DEPC-treated H_2_O was added. Reverse transcription of the mRNA to template cDNA was completed with the transcription RT Kit (TaKaRa Biotech Co., Ltd., Dalian, China). Two nanograms of total RNA were analyzed by real-time PCR with SYBR Green to assess *MMP-3 *expression, and β-actin as housekeeper. PCR primers pairs (Bio Teke Co., Ltd., Beijing, China) used were: *MMP-3*, forward 5'-GGGAAGCTGGACTCGAACACT-3', reverse 5'-TGAGCAGCAACCAGGAATAGG-3'; *β-actin*, forward 5'-CCCATCTATGAGGGTTACGC-3', reverse 5'-TTTAATGTCACGCACGATTTC-3'. Expression values of *β-actin *for each group were averaged and used as a denominator to determine the relative expression level of *MMP-3*.

### Statistical analysis

Results are expressed as the mean ± standard deviation. Statistical analysis was carried out using a one-way ANOVA and Tukey's test for *post hoc *analysis with significance set at *P *< 0.05.

## Results

### Histological evaluation

**Figure 1:**
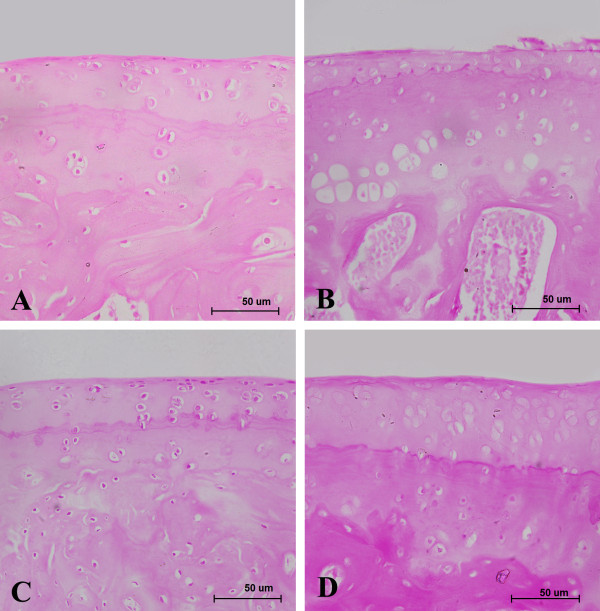
**Histological photographs of the representative cartilage tissues in four groups with safranin-O staining**. Surface regularities and arranged chondrocytes were found in SED group **(A)**. However, surface irregularities, cell cloning and moderate reduction in the safranin-O staining were detected in the HIR group **(B)**. Smooth surface, disarranged chondrocytes and reduction in the safranin-O staining were displayed in HIRI group **(C)**. Compared with the HIRI1 group, lesser reduction in Safranin-O staining was detected in the HIRI2 group (D). scale bar = 50 μm.

The histological appearance of cartilage sections in the SED group demonstrated the normal structure of Wistar rat knee cartilage and subchondral bone (Figure 1A). In contrast, histological changes of surface irregularities, cell cloning and moderate reduction in the safranin-O staining were found in the HIR group (Figure 1B). With the application of MMP-3 inhibitor, the surface became smooth. However, reduction of the safranin-O staining was also detected in the HIRI1 and HIRI2 groups (Figure 1 C, D).

**Figure 2:**
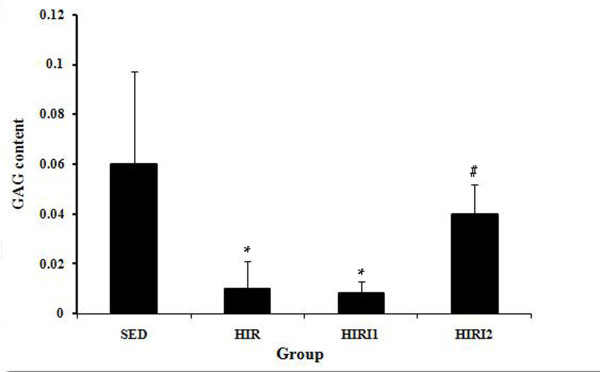
**The results of GAG content in each group**. Significantly lower GAG content in the HIR group was found in comparison with that in the SED group. A significant difference was found between the HIRI2 group and the HIR group. * *P *< 0.05 compared to SED group; ^# ^*P *< 0.05 compared to HIR group.

Figure 2 shows the results of GAG content in each group. GAG content in HIR group (0.010 ± 0.011) was significantly lower than in the SED group (0.060 ± 0.037). The GAG content in the HIRI1 group (0.008 ± 0.005) was similar to that in the HIR group. However, a significant increase was found in the HIRI2 group (0.040 ± 0.012), compared to the HIR group, and no statistical difference was found between the HIRI2 and SED groups.

**Figure 3:**
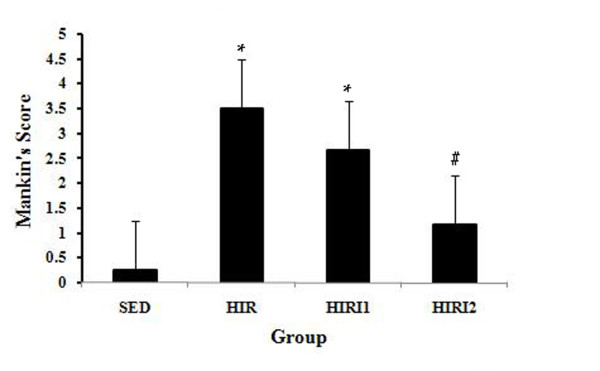
**Mankin's score in each group**. There was a significant increase in the HIR group compared to the SED group. However, compared with the HIR group, Mankin's scores decreased in the HIRI1 group and in the HIRI2 group, and a statistically significant difference was found in the HIRI2 group. * *P *< 0.05 compared to the SED group; ^# ^*P *< 0.05 compared to the HIR group.

Mankin's score in each group is shown in Figure 3. The score in the HIR group (3.500 ± 1.049) was higher than in the SED group (0.250 ± 0.500), which suggested that mild cartilage degradation was induced by six weeks of high intensity running. However, compared to the HIR group, Mankin's scores were lower in the HIRI1 group (2.667 ± 1.033) and the HIRI2 group (1.167 ± 0.753), and a statistically significant difference was found between the HIRI2 and HIR groups. No statistical difference was found between the SED and HIRI2 groups.

### Serum MMP-3 level

**Figure 4:**
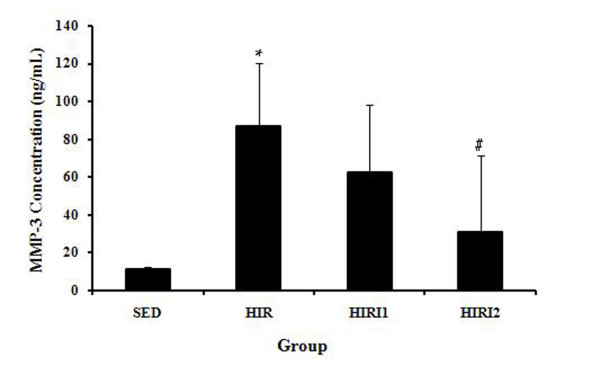
**The results of serum MMP-3 level in all groups**. A significantly higher serum MMP-3 level was found in the HIR group than that in the SED group. However, serum MMP-3 level decreased with the treatment of the MMP-3 inhibitor. Statistical significance was found in the HIRI2 group compared with the HIR group. * *P *< 0.05 compared to the SED group; ^# ^*P *< 0.05 compared to the HIR group.

Figure 4 shows the results of serum MMP-3 level in the four groups. The level in the SED group (11.437 ± 0.920 ng/mL) was significantly lower than in the HIR group (86.850 ± 33.158 ng/mL). However, the serum MMP-3 level decreased with the treatment of the MMP-3 inhibitor. Statistical significance was between the HIRI2 group (30.749 ± 40.416 ng/ml) and the HIR group, but not between the HIRI2 group and SED group.

### Immunohistochemistry

**Figure 5:**
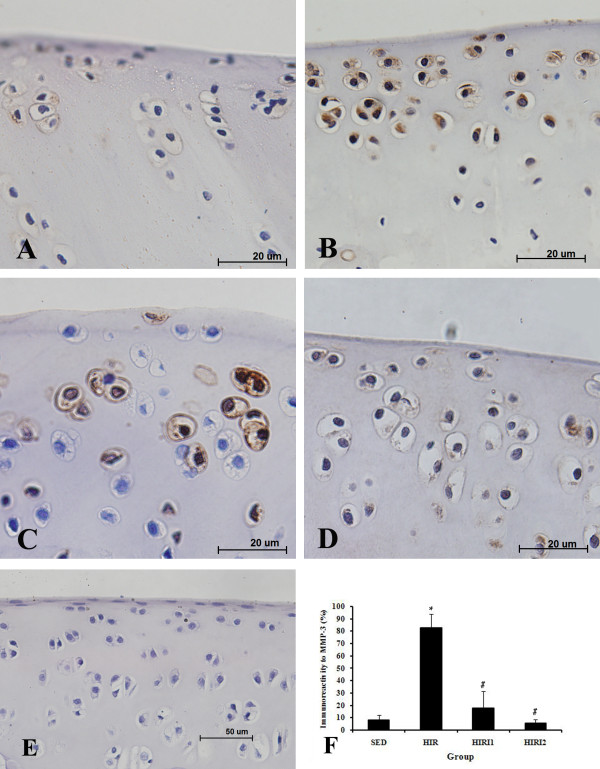
**Immunohistochemical staining of MMP-3 and the percentage of MMP-3 positive chondrocyte in all groups (**A**: SED group; **B**: HIR group; **C**: HIRI1group; **D**: HIRI2 group; **E**: negative control)**.* *P *< 0.05 compared to SED group; ^# ^*P *< 0.05 compared to HIR group.

Immunostaining revealed detectability for MMP-3 of the chondrocytes in cartilage sections. Figure 5 shows the immunohistochemical staining of MMP-3 and the percentage of MMP-3 positive chondrocyte in all groups. In the SED group, 8.35 ± 4.063% of all visible chondrocytes showed immunoreactivity to MMP-3, the ratio rose significantly to 82.53 ± 11.505% in the HIR group. Nevertheless, the ratios decreased significantly to 17.68 ± 13.789% (HIRI1 group) and 5.59 ± 2.977% (HIRI2 group). There was no statistical difference between the HIRI2 group and the SED group.

**Figure 6:**
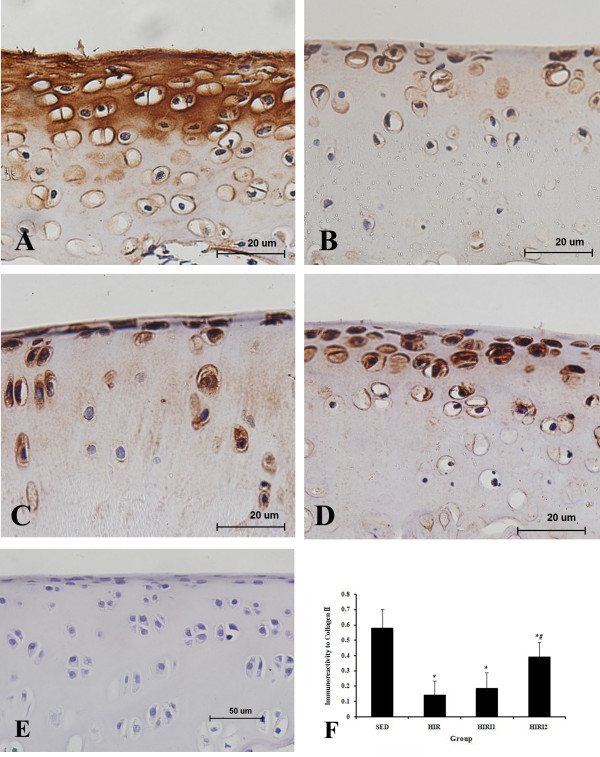
**Immunohistochemical staining of collagen type II and the content in all groups (**A**: SED group; **B**: HIR group; **C**: HIRI1group; **D**: HIRI2 group; **E**: negative control)**. * *P *< 0.05 compared to SED group; ^# ^*P *< 0.05 compared to HIR group.

Immunohistological analysis for collagen type II was performed in all sections, and the content of collagen type II in each group is shown in Figure 6. The collagen content in the SED group (0.577 ± 0.123) was significantly lower than in the HIR group (0.142 ± 0.093). With the application of the MMP-3 inhibitor, the collagen type II content increased slightly in the HIRI1 group (0.186 ± 0.100), and significantly in the HIRI2 group (0.391 ± 0.096). However, the collagen content in HIRI2 group was still significantly lower than in the SED group.

### mRNA gene expression of *MMP-3*

**Figure 7:**
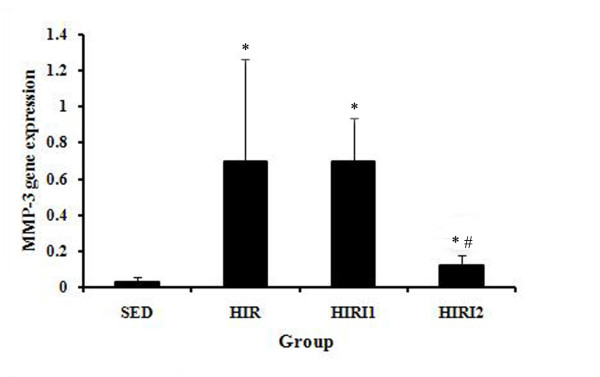
***MMP-3 *gene expression in each group**. There was a statistically significant increase in mRNA gene expression of *MMP-3 *in the HIR group compared to the SED group. A decrease in mRNA expression of *MMP-3 *was found after the treatment with *MMP-3 *inhibitor with a statistical difference only in the HIRI2 group. * *P *< 0.05 compared to SED group; ^# ^*P *< 0.05 compared to HIR group.

As shown in Figure 7, a statistically significant increase of mRNA gene expression of *MMP-3 *in the HIR group (0.697 ± 0.567) was found in comparison to the SED group (0.030 ± 0.026). A decrease of mRNA expression of *MMP-3 *was found after the treatment with *MMP-3 *inhibitor (0.694 ± 0.243 for the HIRI1 group, and 0.121 ± 0.056 for the HIRI2 group); however, only the difference between the SED and HIRI2 groups was considered significant.

## Discussion

Mechanical loading of articular cartilage is essential to regulate the metabolic activity of chondrocytes, and is required for maintaining normal ECM properties. However, it is generally believed that excessive mechanical loading contributes to the degeneration of cartilage and the onset of OA [20]. Rats running 500 m/day in a running wheel for 12 weeks showed local softening of the articular cartilage and decreased GAG content [7]. Early degeneration of cartilage was observed in rats running 1,500 m/day for 10 weeks at a 5° incline on the treadmill [8]. In this current study, rats running 1,500 m/day for six weeks at a 12° incline on the treadmill also showed early degeneration of cartilage. This indicated that during high intensity running, excessive running load was applied on cartilage in the knee joint, which led to cartilage degradation.

Among the many cellular activities reported to be subject to mechanical regulation are the expression and activation of MMPs *in vivo *and *in vitro *[21]. It was reported that chondrocytes respond to a range of mechanical loading conditions through diverse metabolic responses, including MMPs synthesis [13-15,22]. Various mechanical loading may encourage different MMPs expression in cartilage chondrocytes [17]. In particular, Pap *et al*. described that load-dependent running increased the immunoreactivity of MMP-3 in a rat model [7]. In this current study, mild cartilage degradation was found in rats with high-intensity running exercise for six weeks, which corresponded with a significant increase of MMP-3 activity and gene expression. To further confirm the important role of MMP-3 in cartilage degradation, intra-articular injection of MMP-3 inhibitor I was administered. Our findings suggested that the cartilage catabolic change could be retarded by the use of MMP-3 inhibitor I. The results also demonstrated that MMP-3 may act as the key mediator of cartilage degradation, particularly as the result of excessive running.

Along with MMP-3 activity and gene expression in cartilage, the serum MMP-3 level was found to be significantly higher in this study following excessive running for six weeks. In human studies, an elevated serum MMP-3 level has been observed in subjects with joint injury [23] and OA [24-26]; serum MMP-3 level may predict the rate of cartilage loss in an OA joint [27]. Interestingly, our results indicated that serum MMP-3 level decreased after intra-articular MMP-3 inhibitor treatment. The findings of the reduction in *MMP-3 *gene expression in the current study demonstrated that intra-articular MMP-3 inhibitor treatment could prevent the continuous synthesis of MMP-3. Therefore, the decrease of serum MMP-3 level after treatment could be the results of the reduction of MMP-3 molecules synthesized and/or released from the joint after the treatment. However, as a systemic OA biomarker, the serum level of MMP-3 reflected that the changes not only occur in the signal joint, but also in other joints. This may partially explain the finding in our study that the serum MMP-3 level in the HIRI2 group was still approximately two-fold higher than that in the control group.

In this current study, MMP-3 levels, both in serum and cartilage, increased significantly in rats with high-intensity running for six weeks, which was mirrored with the loss of proteoglycan and collagen II. More importantly, such changes in GAG and collagen II induced by high intensity running was reversed by MMP-3 inhibitor treatment, which also implied the requirement of MMP-3 in regulating proteoglycan and collagen II homeostasis. It was, therefore, not surprising that MMP-3 expression corresponded to the appearance of GAG loss in cartilage following high-intensity running, and could be suppressed by the MMP-3 inhibitor. Proteoglycans provide elasticity and lubricity to the joint surface. Aggrecan degradation is an early sign of ECM destruction in osteoarthritic cartilage. On the other hand, type II collagen content was found reduced significantly followed by high-intensity running, which may be explained by the fact that MMP-3 contributed indirectly to the breakdown of type II collagen by activating other MMPs [28]. Collagen fibrils form a meshwork that provides tensile resistance of the cartilage. Tensile failure of the collagen meshwork has been proposed as an important mechanism of failure of articular cartilage [29].

Our results showed that the degradation of cartilage ECM was induced by excessive loading from running in a rat model. *In vitro *evidence suggested that overloading may also lead to chondrocyte death [30,31]. In human OA cartilage, higher numbers of apoptotic chondrocytes were found than those in normal cartilage, suggesting that apoptosis plays an important role in the development of OA [32,33]. In a literature review, Kuht *et al*. indicated that cell death and extracellular matrix degradation are linked and contribute to the chronic matrix remodeling process that characterizes OA [34]. As such, it is assumed that the number of apoptotic chondrocytes may increase following excessive running loading, which contributes to the matrix degradation demonstrated in our study. However, to understand the extent of cell death in this model, as well as whether the MMP-3 inhibitor protects against cell death, further investigations using the TUNEL technique are required.

Due to the important roles in the pathology of cartilage matrix degradation, MMPs have long been considered excellent potential treatment for OA, and inhibition of their activities has proven to be efficacious in a variety of models of experimentally induced as well as spontaneously occurring OA [35-38]. Mainly because of the musculoskeletal side effects caused by the use of broad-spectrum MMP inhibitors, current drug development strategies for treatment of OA are focused on inhibition of specific MMPs implicated in the disease process [39-41]. In this current study, intensity running would initiate the expression and activation of MMP-3, leading to catabolism of ECM in cartilage; such degradation could be retarded by the use of MMP-3 selective inhibitor at appropriate dosages. As such, intra-articular injection of MMP-3 inhibitor appears to be an effective treatment for retarding the cartilage degradative changes particularly induced by excessive running. However, as such inhibitive effects were found to be dosage-dependent in this current study, further investigations are required for the optimal dosage of MMP-3 inhibitor.

## Conclusions

Mild cartilage degradation was observed in rats after high-intensity running for six weeks, and MMP-3 clearly acted as the key mediator of this catabolic change under such mechanical conditions. A chrondroprotective effect was provided by intra-articular injection of MMP-3 inhibitor, which not only confirmed the important role of MMP-3 in the changes of articular cartilage induced by excessive running load, but also suggested a possible effective treatment for retarding such osteoarthritic changes.

## Abbreviations

ECM: extracellular matrix; ELISA: enzyme-linked immunosorbent assay; GAG: glycosaminoglycan; HIR: high-intensity running; MMPs: matrix metalloproteinases; OA: osteoarthritis; PCR: polymerase chain reaction; SED: sedentary control.

## Competing interests

The authors declare that they have no competing interests.

## Authors' contributions

GN conceived of the study, participated in its design and wrote most of the manuscript. LZ performed most of the experiments and analyzed data. MG carried out the immunohistochemistry and PCR evaluation. LL and YZ helped to analyze data and draft the manuscript. YP participated in the study design and coordination. All authors read and approved the final manuscript.
